# Royal College of Surgeons: Annual Meeting

**Published:** 1915-12-04

**Authors:** 


### ROYAL COLLEGE OF SURGEONS: ANNUAL MEETING.

The annual meeting of the Fellows and Members of the Royal College of Surgeons is not even at the best of times a very impressive event, and this year it has been deprived by military claims of one of its most critical and competent voices. Time and again the same small and select assembly meets to examine the conduct of the Council, to ask the President embarrassing questions, and to pass a resolution demanding the direct representation of the members on the governing body. These annual experiences seem neither to tire the perseverance of the reformers, nor to influence the Council, nor to arouse any practical interest in the great body of the members. Nobody appears to be either the better or the worse for the performance, and after the curtain has been duly rung down silence reigns supreme for another year. Some credit is certainly due on the score of patience to those who continue undismayed through so discouraging a series of experiences. But it may be suggested that their true grievance is less against the Council than against their colleagues. The Council is in possession, and has a long tradition in defence of the fashion in which it discharges its responsibilities. Moreover, with rare exceptions, the Fellows of the College who, speaking generally, are particularly interested in the maintenance of the highest surgical standards, support the Council, and though it may be said that they do so from interested motives, their votes have great weight both with the public and with the official world. If the position is ever to be carried against the authorities some more powerful agency than an annual outburst of condemnatory eloquence must be secured, and it may be doubted whether any such agency is available so long as the great majority of the members of the College remain indifferent to the efforts of their heroic colleagues of the annual meeting.

On the face of things it does of course seem absurd that those who bear the title of " Members " of a surgical college should have no voice in the conduct of its affairs. Yet it cannot be denied that as a matter of fact the great majority of the members are concerned with surgery not in a special, but only in a general and indirect sense. For all practical purposes the diploma which they hold is merely a licence to practise, and the great majority of them have other cares than the craft to which the College is devoted. Even when this consideration is allowed it is difficult to deny that they may reasonably claim some opportunity of presenting their special point of view in relation to the policy of the College, and unless they have some measure of representation this end is not secured. But we very much doubt whether either surgery or the profession generally would be helped by making the Council an organisation charged with the redress of grievances rather than with the interests of the science and art of surgery. There seems the less need for such a change seeing that there are already in existence agencies whose activities are devoted to medico-political ends. In any event, the scanty advance guard of the annual meeting must obviously command the support of the general body before it can hope to storm the official trenches.

It is interesting to note the effect of names in this discussion. While the College of Surgeons calls its diplomates "members," the College of Physicians uses the strict term " licentiate." In the one case there is a suggestion of rights, in the other of duties. It is significant that while the surgical College suffers an annual attack, no one disturbs the serene slumber of the sister institution. Rumour has it that the attempt was once made, but the name of the hero has perished from among men.

